# *Porphyromonas gingivalis* GroEL Induces Osteoclastogenesis of Periodontal Ligament Cells and Enhances Alveolar Bone Resorption in Rats

**DOI:** 10.1371/journal.pone.0102450

**Published:** 2014-07-24

**Authors:** Feng-Yen Lin, Fung-Ping Hsiao, Chun-Yao Huang, Chun-Ming Shih, Nai-Wen Tsao, Chien-Sung Tsai, Shue-Fen Yang, Nen-Chung Chang, Shan-Ling Hung, Yi-Wen Lin

**Affiliations:** 1 Division of Cardiology, Taipei Medical University Hospital, Taipei, Taiwan; 2 Cardiovascular Research Center, Taipei Medical University Hospital, Taipei, Taiwan; 3 Department of Internal Medicine, School of Medicine, College of Medicine, Taipei Medical University, Taipei, Taiwan; 4 Institute of Oral Biology, National Yang-Ming University, Taipei, Taiwan; 5 Division of Cardiovascular Surgery, Taipei Medical University Hospital, Taipei, Taiwan; 6 Division of Cardiovascular Surgery, Tri-Service General Hospital, National Defense Medical Center, Taipei, Taiwan; 7 Department of Dentistry, National Yang-Ming University, Taipei, Taiwan; 8 Department of Stomatology, Taipei Veterans General Hospital, Taipei, Taiwan; University of California, Merced, United States of America

## Abstract

*Porphyromonas gingivalis* is a major periodontal pathogen that contains a variety of virulence factors. The antibody titer to *P. gingivalis* GroEL, a homologue of HSP60, is significantly higher in periodontitis patients than in healthy control subjects, suggesting that *P. gingivalis* GroEL is a potential stimulator of periodontal disease. However, the specific role of GroEL in periodontal disease remains unclear. Here, we investigated the effect of *P. gingivalis* GroEL on human periodontal ligament (PDL) cells *in vitro*, as well as its effect on alveolar bone resorption in rats *in vivo*. First, we found that stimulation of PDL cells with recombinant GroEL increased the secretion of the bone resorption-associated cytokines interleukin (IL)-6 and IL-8, potentially via NF-κB activation. Furthermore, GroEL could effectively stimulate PDL cell migration, possibly through activation of integrin α1 and α2 mRNA expression as well as cytoskeletal reorganization. Additionally, GroEL may be involved in osteoclastogenesis via receptor activator of nuclear factor κ-B ligand (RANKL) activation and alkaline phosphatase (ALP) mRNA inhibition in PDL cells. Finally, we inoculated GroEL into rat gingiva, and the results of microcomputed tomography (micro-CT) and histomorphometric assays indicated that the administration of GroEL significantly increased inflammation and bone loss. In conclusion, *P. gingivalis* GroEL may act as a potent virulence factor, contributing to osteoclastogenesis of PDL cells and resulting in periodontal disease with alveolar bone resorption.

## Introduction

Periodontitis is a bacterially induced inflammatory disease that destroys the four periodontal tissue structures: gingiva, cementum, alveolar bone, and the periodontal ligament. The periodontal connective tissue is degraded first due to the hyperinflammatory reaction, and the underlying alveolar bone is then destroyed, ultimately resulting in tooth loss if the disease is poorly controlled. Thus far, several bacterial species have been reported to be associated with periodontitis, among them *Porphyromonas gingivalis*, an anaerobic gram-negative bacterium that is strongly associated with disease progression [1], [2]. It has been postulated that *P. gingivalis* contributes to tissue and bone destruction in periodontitis by releasing a set of virulence factors including lipopolysaccharide (LPS) and gingipains [3], [4]. Additionally, a previous paper has showed that sera from periodontitis patients test positive for *P. gingivalis* GroEL protein in western immunoblot assays, indicating the presence of an immune response to *P. gingivalis* GroEL in periodontitis patients [5]. Furthermore, the antibody titer to *P. gingivalis* GroEL is significantly higher in periodontitis patients than in healthy control subjects [6], and periodontal treatment can significantly decrease the level of anti-*P. gingivalis* GroEL antibodies in sera [7]. Additionally, a positive relationship has been observed between levels of salivary IgA directed against GroEL and periodontal disease severity [8], and a *P. gingivalis* GroEL protein vaccine reduces bacterially induced multiple periodontopathogenic alveolar bone loss [9], indicating that *P. gingivalis* GroEL is a potential immunodominant antigen in patients with periodontitis and may contribute to pathogenic processes.

GroEL, a homologue of heat shock protein 60 (HSP60), belongs to the heat shock protein 60 family and has an important role in the folding of newly synthesized proteins, preventing misfolding and aggregation. However, GroEL is also widely recognized as an important molecule in various bacterial infections and autoimmune diseases [10], [11]. Several studies have reported that some bacterial HSPs stimulate the production of pro-inflammatory cytokines in human monocytes [12]–[14] as well as the upregulation of adhesion molecule expression [15], [16]. It is well known that *Campylobacter rectus* GroEL and *Aggregatibacter actinomycetemcomitans* GroEL can stimulate the production of interleukin-6 (IL-6) or IL-8 by human gingival fibroblasts and human gingival epithelial cells [17]–[19]. *P. gingivalis* GroEL is also able to stimulate nuclear factor-kappa B (NF-κB) transcriptional activity, which is significantly inhibited by anti-human Toll-like receptor 2 (hTLR2) and anti-human Toll-like receptor 4 (hTLR4) antibodies in THP-1 cells, suggesting that *P. gingivalis* GroEL induces its intracellular signaling cascade in THP-1 cells via the TLR2 or TLR4 receptors [20]. The studies described above strongly suggest that the GroEL from periodontopathogenic bacteria may possess biological activities that are involved in the progression of periodontal disease. Although *P. gingivalis* GroEL is suggested to be a potent stimulator of inflammatory cytokines in periodontal disease, its virulent effects are not yet understood in detail. Thus, the aim of this study was to investigate the responses underlying the virulence of *P. gingivalis* GroEL in periodontal ligament (PDL) cells *in vitro* and in rat periodontal tissues *in vivo*.

## Materials and Methods

### Ethics Statement and Culture of Periodontal Ligament Cells

The Institutional Review Board (Taipei Veterans General Hospital-Joint Institutional Review Board) approved this study (Protocol No.: V100C-053), and all volunteers gave written informed consent prior to all procedures. Periodontal ligament (PDL) cells, a major component of the tooth-supporting tissue that plays an important role in maintaining periodontal tissue homeostasis, were used in this study. Primary human PDL cells were isolated from healthy human PDL tissue that was obtained from premolars extracted for orthodontic reasons by previously described methods [21], [22]. To avoid contamination from the gingiva and the dental pulp, only the middle of the tooth root was collected. The cells were maintained in Dulbecco’s modified Eagle medium (DMEM; Gibco-BRL, Rockville, MD, USA) containing 10% (v/v) heat-inactivated fetal bovine serum (FBS; HyClone Laboratories, Logan, UT, USA) and 1×(v/v) penicillin/streptomycin (Sigma-Aldrich, St. Louis, MO, USA) at 37°C in a humidified atmosphere of 95% air and 5% CO_2_. PDL cells between the 3rd and 7th passages were used in the present study.

### Construction of *P. gingivalis* GroEL Expression Vector

*P. gingivalis* genomic DNA (ATCC No. 33277) was extracted using an EasyPure Genomic DNA mini kit (Bioman Scientific Co., Taipei, Taiwan). The region containing the GroEL open reading frame was originally PCR amplified using 100 ng of *P. gingivalis* genomic DNA as a template, 0.2 mM dNTPs, 1 µM of each gene-specific primer and 1 U Pfu DNA polymerase (Promega, Madison, WI, USA) with the following program: one cycle of 95°C for 5 min; 38 cycles of 95°C for 45 sec, 68°C for 45 sec, and 72°C for 2 min; 1 cycle of 68°C for 45 sec and 72°C for 10 min; and a final incubation at 72°C for 10 min with 1 U Taq DNA polymerase.

The GroEL-specific forward and reverse primers we used in the PCR are shown in Table 1. The amplified ∼1.7 K GroEL cDNA fragment was then cloned into the pCR2.1-TOPO vector (Invitrogen, Carlsbad, CA, USA) for sequencing. Subsequently subcloned the correct in-frame using the EcoRI sites of the pGEX-5X-1 expression vector, which contains a GST tag sequence in the 5′ end of the multiple cloning site, (GE Healthcare Amersham Biosciences, Piscataway, NJ, USA) for expression in *E. coli*.

**Table 1:** PCR primers used for the amplification of genes.

Gene name	Forward primer (5′→3′)	Reverse primer (5′→3′)
GroEL	ACT ATG GCA AAA GAA ATC AAA TTC GAT ATG	CCT GTT CGC ACC GAT GTT TAC ATC
IL-6	GTA GCC GCC CCA CAC AGA	CAT GTC TCC TTT CTC AGG GCT G
IL-8	ATA AAG ACA TAC TCC AAA CCT TTC CAC	AAG CTT TAC AAT AAT TTC TGT GTT GGC
ALP	TGG ATC ACA GCA CAT CAT CAG AGC AG	CTC ACT TTA TGG GAA CCA GAT GG
RANKL	CTG CCA TCC TGT ATG GCA ATG	AGA CTG CGC CTG GTA GTT GTT G
Integrin α1	TGC CAT TAT GGG TCA TCC TGC TG	CAC ATA TTT GAG GCA AAC CTG AGG
Integrin α2	CAT CAA CGT TCC AGA CAG TAC AGC	GCT AAC AGC AAA AGG ATT CCA GC
Integrin β1	CTG GTG TGG TTG CTG GAA TTG TTC	CCT CAT ACT TCG GAT TGA CCA CAG
GAPDH	TCA CCA CCA TGG AGA AGG C	GCT AAG CAG TTG GTG GTG CA

IL, interleukin; ALP, alkaline phosphatase; RANKL, receptor activator of nuclear factor kappa-B ligand; GAPDH, glyceraldehyde-3-phosphate dehydrogenase.

### Purification of GST-tagged Rrecombinant *P. gingivalis* GroEL

BL21 cells were transformed with either pGEX-5X-1 expression vector or pGEX-5X-1- *P. gingivalis* GroEL expression vector, after which GST, which was used as a control in all experiments in this study, or the GST-tagged recombinant GroEL protein was purified, respectively. Briefly, BL21 cells containing the expression plasmids were grown overnight at 37°C in 2 mL LB medium supplemented with 100 µg/mL ampicillin. Next, 1.25 mL of each overnight culture was transferred into 100 mL LB/ampicillin medium and grown at 37°C to an A600 of 0.6–0.8 (approximately 2 h). Protein expression was induced by adding IPTG to a final concentration of 1 mM and incubating at 30°C for 6 h. Bacteria were pelleted by centrifugation for 10 min at 8000 rpm, and the proteins were extracted under native conditions according to the GST Gene Fusion System manufacturer’s instructions. Finally, the recombinant proteins were purified using an elution buffer containing 50 mM Tris-HCl and 10 mM reduced glutathione (pH 8.0). GST and GST-tagged *P. gingivalis* GroEL proteins were quantified using the Bio-Rad Protein Assay (Bio-Rad, Hercules, CA, USA). The purity was further confirmed using a Coomassie blue-stained SDS polyacrylamide gel, and protein identity was confirmed by immunoblotting with an anti-GST antibody (GE Healthcare Amersham Biosciences, Piscataway, NJ, USA).

### MTT Aassay and Eenzyme-linked Iimmunosorbent Aassay (ELISA)

The cytotoxicity of the recombinant GroEL protein was analyzed by the 3-(4,5-dimethylthiazol-2-yl)-2,5-diphenyl tetrazolium bromide (MTT) assay. Human PDL cells were grown in 96-well plates at a density of 1.6×10^4^ cells/well and incubated with serum-free medium (0 µg/mL; control), 25–100 µg/mL GroEL or 100 µg/mL GST for 24–72 h. Subsequently, 0.5 µg/ml MTT was added to each well, and incubation was continued at 37°C for an additional 4 h. Finally, the MTT solution was discarded, dimethyl sulfoxide (DMSO) was added to each well, and the absorbance was recorded at 530 nm using a DIAS Microplate Reader (Dynex Technologies Headquarters, Sullyfield Circle Chantilly, VA, USA). The effects of GroEL on cytokine production in PDL cells were measured by enzyme-linked immunosorbent assay (ELISA). Human PDL cells were seeded in 24-well plates at a density of 10^6^ cells/well and then treated with 0–50 µg/mL GroEL or 50 µg/mL GST in 500 µL serum-free medium for 24 h. The culture medium was collected for quantification of IL-6 and IL-8 levels using the DuoSet ELISA development kits (R&D Systems Inc., McKinley Place N.E., Minneapolis, USA.), and the absorbance was recorded using a TECAN Sunrise ELISA Reader (Tecan Group Ltd., Männedorf, Switzerland).

### Cell Wound-healing Assay

Because PDL cells have been reported to play critical roles in wound healing and regeneration of periodontal tissue [23], [24], we performed a wound healing assay. The migratory ability of GroEL-pretreated PDL cells was assayed using a monolayer denudation assay [25]. Confluent PDL cells (3×10^5^/well) in a 6-well plate were cultured with serum free medium (0 µg/mL; control), 10–50 µg/mL GroEL or 50 µg/mL GST for 24 h prior to wound scraping using a 100-µL pipette tip that denuded a strip of the monolayer that was 300 µm in width. The cultures were washed twice with PBS and incubated with medium supplemented with 5% FBS, and the rate of wound closure was observed 24 h after wound scraping. The width of the gap was measured under the 4× phase objective of a light microscope (OLYMPUS IX71, Olympus Imaging America Inc., Center Valley, PA, USA), monitored with a CCD camera (Macro FIRE 2.3A), and captured with a video graphic system (Picture Frame Application 2.3 software).

### Quantitative Real-time Polymerase Chain Reaction (qPCR)

PDL cells have the ability to regulate osteoclast differentiation through activation and secretion of RANKL, an osteoblast-activated gene [26]. ALP, a factor associated with calcification and osteogenesis, is upregulated in PDL cells expressing an osteoblastic phenotype [27]. Additionally, integrins play a major role in mediating PDL cells migration. Hence, the mRNA levels of integrin α1, integrin α2, integrin β1, RANKL and ALP mRNA in *P. gingivalis* GroEL-stimulated PDL cells was analyzed using quantitative real-time PCR. Total RNA was isolated using TRIzol Reagent (Invitrogen, Carlsbad, CA, USA) according to the manufacturer’s instructions. First-strand cDNA synthesis was carried out using SuperScript III First-Strand Synthesis System Kit with 1 µg of total RNA (Invitrogen, Carlsbad, CA, USA). The cDNA samples were then used for qPCR, performed in an ABI StepOnePlus Real-Time PCR System (Applied Biosystems Inc., Lincoln Centre Drive Foster City, CA, USA). SYBR Green qPCR Master Mix (2×) and ROX (Fermentas, Thermo Fisher Scientific Inc., Rockford, IL, USA) were used for the amplification reactions, with each primer pair listed in Table 1 added separately and 40 cycles in qPCR amplification were used. To normalize the Ct values, the housekeeping gene glyceraldehyde-3-phosphate dehydrogenase (GAPDH) was used.

### Immunofluorescent Staining for NF-kB and F-actin

PDL cells seeded on cover slips (4×10^4^ cells) were treated with serum-free medium (0 µg/mL; control), GroEL (25 or 50 µg/mL) or 50 µg/mL GST for 120 min. After washing once, the cells were fixed with 4% paraformaldehyde and permeabilized for 10 min with a 0.1% Triton X-100 solution. Nonspecific antigens were blocked with 2% bovine serum albumin for 30 min and the slides were then stained with either rhodamine-conjugated phalloidin or a mouse anti-NF-κB (sc-8008, Santa Cruz biotechnology, Santa Cruz, CA, USA) antibody. Alexa 488-conjugated anti mouse IgG antibody was used to detect the mouse anti-NF-κB antibody, and nuclei were identified using Hoechst 33258 (Sigma, St. Louis, MO, USA). Images were obtained using a Zeiss LSM 510 Meta confocal microscope (Carl Zeiss MicroImaging Inc., Thornwood, NY, USA).

### Grouping of Animal Experiments

All animals were treated according to protocols approved by the Institutional Animal Care Committee of the National Yang-Ming University (Permit No.: 991241), Taipei, Taiwan. This study was carried out in strict accordance with the recommendations in the Guide for the Care and Use of Laboratory Animals of the National Institutes of Health (NIH Publication No. 85–23, revised 1996). Eighteen 250–300 g adult male Sprague-Dawley rats were purchased from BioLASCO Co. (I-Lan, Taiwan). The animals were divided into 3 groups (n = 6 per group): group 1 was the control, which received 25 µL of endotoxin-free PBS; group 2 received 25 µg of *P. gingivalis* GroEL (1 µg/µL); and group 3 received 25 µg of GST protein (1 µg/µL). The animal model was established as previously described [28], with modification. Briefly, samples were injected into the interdental gingiva at the tongue (mesial) aspect, between the left maxillary first (M1) and second (M2) molar, under isoflurane aspirating anesthesia. The injections, which were performed using 1/2-inch×30 gauge needles (BD, Franklin Lake, NJ, USA) attached to 50 µl syringes (Hamilton company, Reno, NV, USA), were repeated three times per week over a 6-week period.

### Specimen Preparation

Animals were euthanized by CO_2_ overdose, and the intact maxillary jaw and associated structures were fixed in cold 4% paraformaldehyde for 24 h and then stored in 70% ethanol for scanning by micro-CT, after which the maxillary jaws were decalcified for 14 days with daily solution changes. The jaws were then embedded in paraffin and sectioned at 5 microns.

### Micro-computed Tomography Analysis

Micro-computed tomography (CT) was carried out at the Taiwan Mouse Clinic (TMC), Taipei, Taiwan, and quantitation was performed as previously described [29]. The specimens were scanned by micro-CT (SkyScan 1076, Bruker Co., Brussels, Belgium) at a resolution of 35 µm in all spatial dimensions. Transverse scanning initiated at a line between the left maxillary first molar and second molar furcations and continued in the apical (root) direction for 20 successive scan slices, with the outer walls of the scan determined by the root surface. The results were analyzed with the CTan software (Bruker Co., Belgium, China) for the quantification of bone volume and total volume and were represented as the residual bone volume (BV) *per* total volume (TV) for 20 slices [29]. [BV/TV×100] should be always less than 100% because TV includes all of the bone, PDL cells, and the other connective tissues.

### Histomorphometric Analysis and Immunohistochemistry

Five µm sections were first stained with hematoxylin and eosin, and the distance between the cemento-enamel junction and alveolar crest height (CEJ-AJ) was evaluated. Immunohistochemical staining to assess inflammation was performed using anrabbit anti-IL-6 antibody (NB600-1131, Novus, Saint Charles, MI, USA) or rabbit anti-IL-8 antibody (ab7747, Abcam Inc., CA, USA). Additionally, inflammatory infiltration of the gingival connective tissue was assayed by staining with an anti-CD68 antibody (NB100-683, Novus, Saint Charles, MI, USA) to detect macrophages. Antibody stains were developed via DAB (diaminobenzidine tetrachloride)/hydrogen peroxide (Dakocytomation, Produktionsvej, Glostrup, Denmark) reaction. Sections were subsequently counterstained with hematoxylin, dehydrated, and mounted. Finally, the slides were observed using light microscopy.

### Tartrate resistant acid phosphatase (TRAP) histochemical staining

TRAP histochemistry was used to identify active osteoclasts in sections. According to the previously description [30], the five µm the sections were incubated in warmed TRAP staining solution mix which containing 0.1 M sodium acetate anhydrous, 0.05 M L-(+) tartaric acid, 600 µg/mL fast red violet LB salt, and 100 µg/mL naphthol AS-MX phosphate substrate for 45 minutes. Sections were subsequently counterstained with 0.02% Fast Green, and mounted. Finally, the slides were observed using light microscopy.

### Statistical Analyses

Values were expressed as means ± standard error of the mean (SEM). SigmaStat software version 3.5 (Systat Software Inc., Chicago, IL, USA) was used for statistical analysis. Statistical evaluation was performed using Student’s t-test and one- or two-way ANOVA followed by Dunnett’s test. A probability value of p<0.05 was considered significant.

## Results

### *P. gingivalis* GroEL Induces IL-6 and IL-8 Production in PDL Cells, Potentially via NF-kB Activation

**Figure 1:**
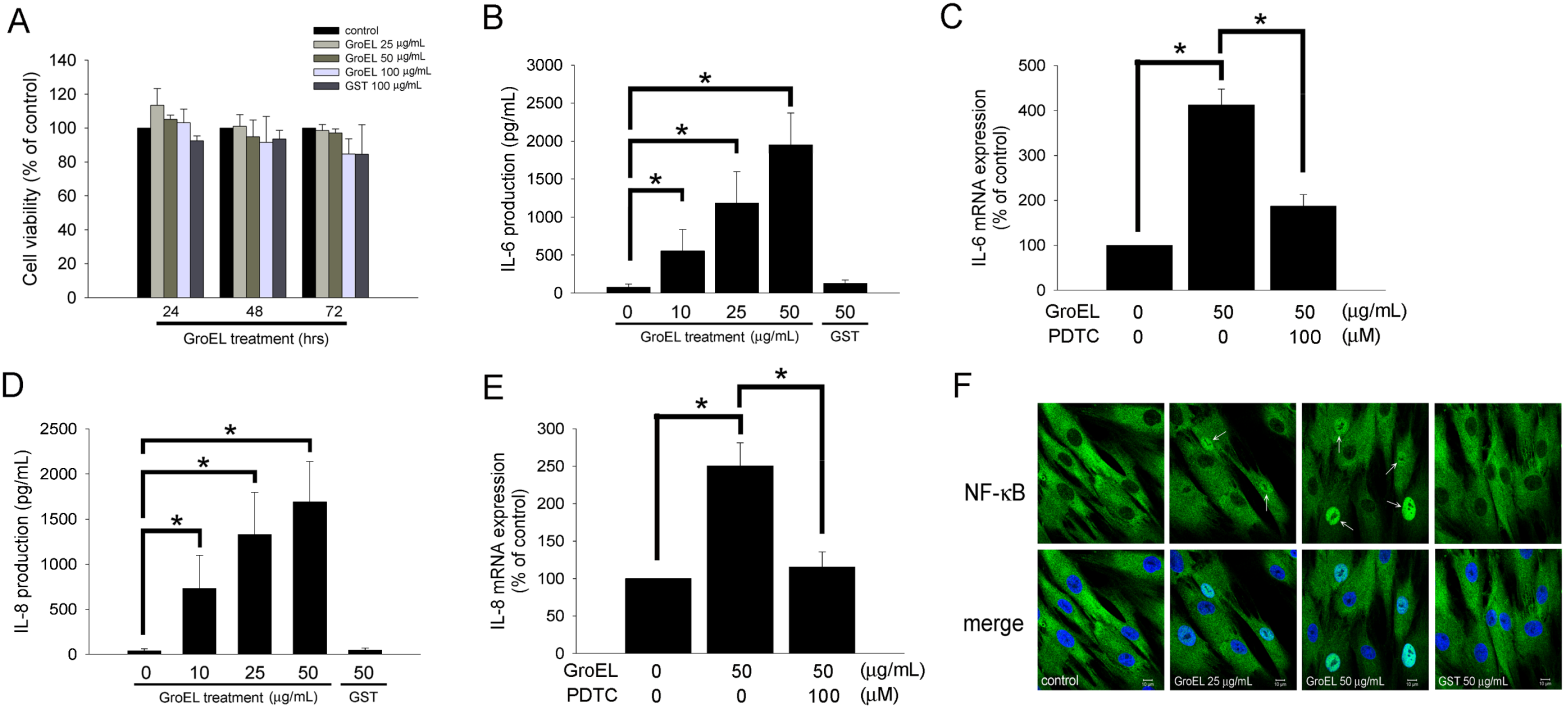
*P. gingivalis* GroEL induces IL-6 and IL-8 production in PDL cells, potentially via NF-κB activation. (A) Cellular cytotoxicity of recombinant *P. gingivalis* GroEL was analyzed by MTT assay. PDL cells were treated with serum-free medium (0 µg/mL; control), 25–100 µg/mL GroEL or 100 µg/mL GST for 24–72 h; an MTT assay was performed, and the absorbance was recorded using a microplate reader. (B and D) PDL cells were treated with serum-free media containing 0–50 µg/mL GroEL or 50 µg/mL GST for 24 h. The levels of IL-6 (B) and IL-8 (D) in the culture media were quantified via ELISA assay, and the absorbance was recorded using a microplate reader. (C and E) PDL cells were treated with serum-free medium (0 µg/mL; control), 50 µg/mL GroEL or 100 µM pyrrolidine dithiocarbamate (PDTC) plus 50 µg/mL GroEL for 12 h. The expression of IL-6 (C) and IL-8 (E) mRNA was analyzed using quantitative real-time PCR. Data are expressed as the mean ± SEM of three independent experiments performed in triplicate. *p<0.05 was considered significant. (F) PDL cells grown on slides were exposed to serum-free medium (0 µg/mL; control), GroEL (25 or 50 µg/mL) or 50 µg/mL GST for 120 min and then imaged on a confocal microscope after immunofluorescence staining against NF-κB p65 was performed. The lower images are merged pictures of the upper images with Hoechst staining for nuclei. White arrows indicate activated NF-κB p65 in the nuclei. The scale bar indicates 10 µm.

We first determined the toxicity of recombinant *P. gingivalis* GroEL. The treatment of PDL cells with 25, 50, or 100 µg/mL of GroEL for 24–72 h did not affect PDL cell viability by the MTT assay (figure 1A). As the *P. gingivalis* GroEL protein was produced using a GST-tagged *E. coli* expression system, control groups treated with GST tag protein alone were included in all experiments to exclude the possibility that the observed effects were caused by the GST tag. The effect of *P. gingivalis* GroEL on the expression of IL-6 and IL-8, proinflammatory cytokines that influence bone resorption, was determined. Treatment of PDL cells with 10, 25 and 50 µg/mL of GroEL for 24 h significantly increased the production of IL-6 in a dose-dependent manner by 554.51±281.99, 1182.66±414.17, and 1949.82±633.75 pg/mL, respectively (76.23±41.34 pg/mL in control group; figure 1B), while production of IL-8 was increased by 731.58±365.79, 1131.45±465.72, and 1692.63±446.31 pg/mL, respectively (41.29±20.64 pg/mL in control group; figure 1D). Treatment of PDL cells with 50 µg/mL of GST protein had no effect on cytokine production, indicating that the observed effects were due to GroEL. As transcriptional regulation involving NF-κB activation has been implicated in the inflammatory responses of PDL cells, we analyzed the role of NF-κB and monitored the translocation of NF-κB p65 to the nuclei of PDL cells by quantitative real-time PCR and immunofluorescence staining, respectively. The results showed that treatment with 50 µg/mL of GroEL for 12 h significantly increased IL-6 (figure 1C) and IL-8 (figure 1E) mRNA production. Pretreatment with 100 µM of pyrrolidine dithiocarbamate (an inhibitor of NF-kB) for 30 min may decrease the IL-6 and IL-8 mRNA production in GroEL-stimulated PDL cells. In addition, 25 and 50 µg/mL of GroEL markedly increased NF-κB p65 staining in the nuclei (white arrows) relative to untreated (0 µg/mL) or 50 µg/mL GST-treated cells (figure 1F). These results suggested that *P. gingivalis* GroEL was able to stimulate the production of IL-6 and IL-8, potentially through activation of NF-κB signaling in PDL cells.

### *P. gingivalis* GroEL Increases PDL Cell migration, Potentially Through Activation of Integrin α1 and α2 Expression, as well as Cytoskeletal Reorganization

**Figure 2:**
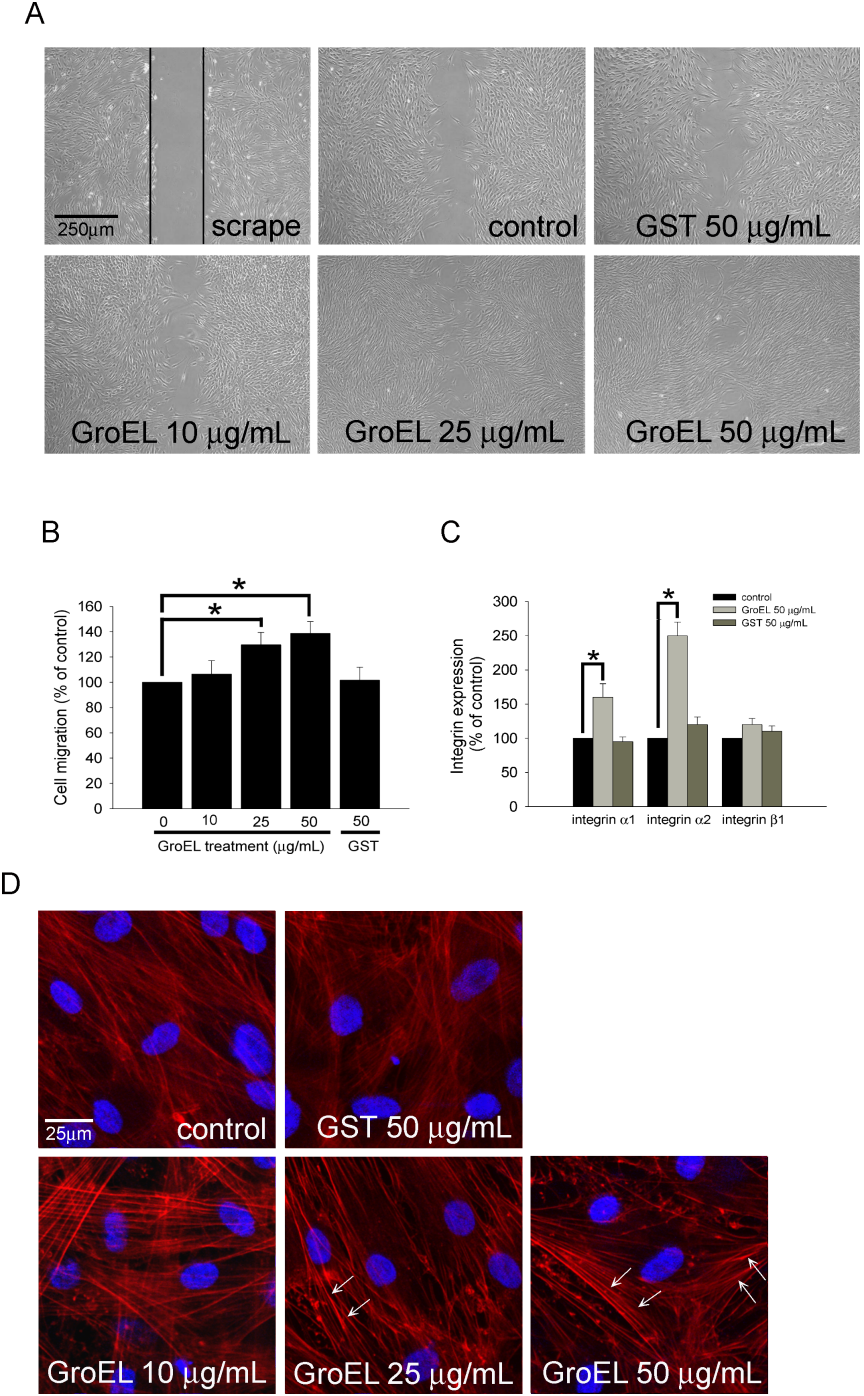
*P. gingivalis* GroEL increases PDL cell migration, possibly through activation of integrin α1 and α2 expression, as well as cytoskeletal reorganization. (A) A wound-healing assay was used to evaluate the effect of GroEL on PDL cell migration. PDL cells were cultured with serum free medium (0 µg/mL; control), 10–50 µg/mL GroEL or 50 µg/mL GST for 24 h before wound scraping using a pipette tip. Images were taken 24 h after wound scraping. PDL cells migrating to the denuded area were counted based on the black base line. (B) PDL cells that migrated into the denuded area were quantified, and the magnitude of PDL cell migration was evaluated by counting the migrated cells in six random areas under high-power microscope fields (×100). (C) PDL cells were treated with serum-free medium (0 µg/mL; control), 50 µg/mL GroEL or 50 µg/mL GST for 12 h. The expression of integrin α1, α2, and β1 mRNA was analyzed using quantitative real-time PCR. Data are expressed as the mean ± SEM of three independent experiments and expressed as the percentage of control. *P<0.05 was considered significant. (D) F-actin was stained with rhodamine-phalloidin, and the staining was evaluated using confocal microscopy at 400× magnification. The visible parallel stress fibers are indicated as white arrows. DAPI was used to identify the nuclei of PDL cells.

As shown in figure 2, treatment of cells with 25 and 50 µg/mL of GroEL for 24 h induced their wound-healing/migratory abilities in a dose-dependent manner relative to the control group (129.7±9.7% and 138.7±9.4% of 0 µg/mL control, respectively) (figure 2A and B). Next, the quantitative real-time PCR was used to determine the expression of integrins α1, α2, and β1 mRNA. The results demonstrated that treatment with 50 µg/mL of GroEL for 12 h significantly increased the mRNA levels of the receptors for collagen, integrin α1 and integrin α2 (160.5±20.5% and 250.3±20.5%, respectively), but had no effect on the expression of integrin β1 mRNA (figure 2C). The cytoskeleton is a key structure that facilitates cellular proliferation and migration [31], therefore, cytoskeletal dynamics in GroEL-treated cells were further examined. PDL cells were treated with 10–50 µg/mL of GroEL for 24 h and were then fixed and stained with rhodamine-conjugated phalloidin, which specifically stains F-actin. Figure 2D shows that control (0 µg/mL) PDL cells with F-actin distributed in a crisscrossing and overlap pattern. Within the cell, the scattering and even spread of F-actin were observed. However, treatment of cells with either 25 or 50 µg/mL of GroEL resulted in prominent phalloidin staining and the appearance of stress fibers that were visible in a parallel pattern, crossing the cell from one side to the other. The phenomena of crisscrossing overlap pattern, scattering, and even spread of F-actin were disappeared. Additionally, GroEL-treated PDL cells displayed peripheral bands, clusters (white arrow) and punctiform congregate of F-actin in the cell margin, indicating the redistribution, assembly, and organization of F-actin in the cytoskeleton. These results indicated that *P. gingivalis* GroEL strongly stimulates PDL cell migration, potentially through activation of integrin α1 and α2 expression, as well as cytoskeletal reorganization.

### *P. gingivalis* GroEL May Be Involved in Osteoclastogenesis via RANKL Activation and ALP Inhibition in PDL Cells

**Figure 3:**
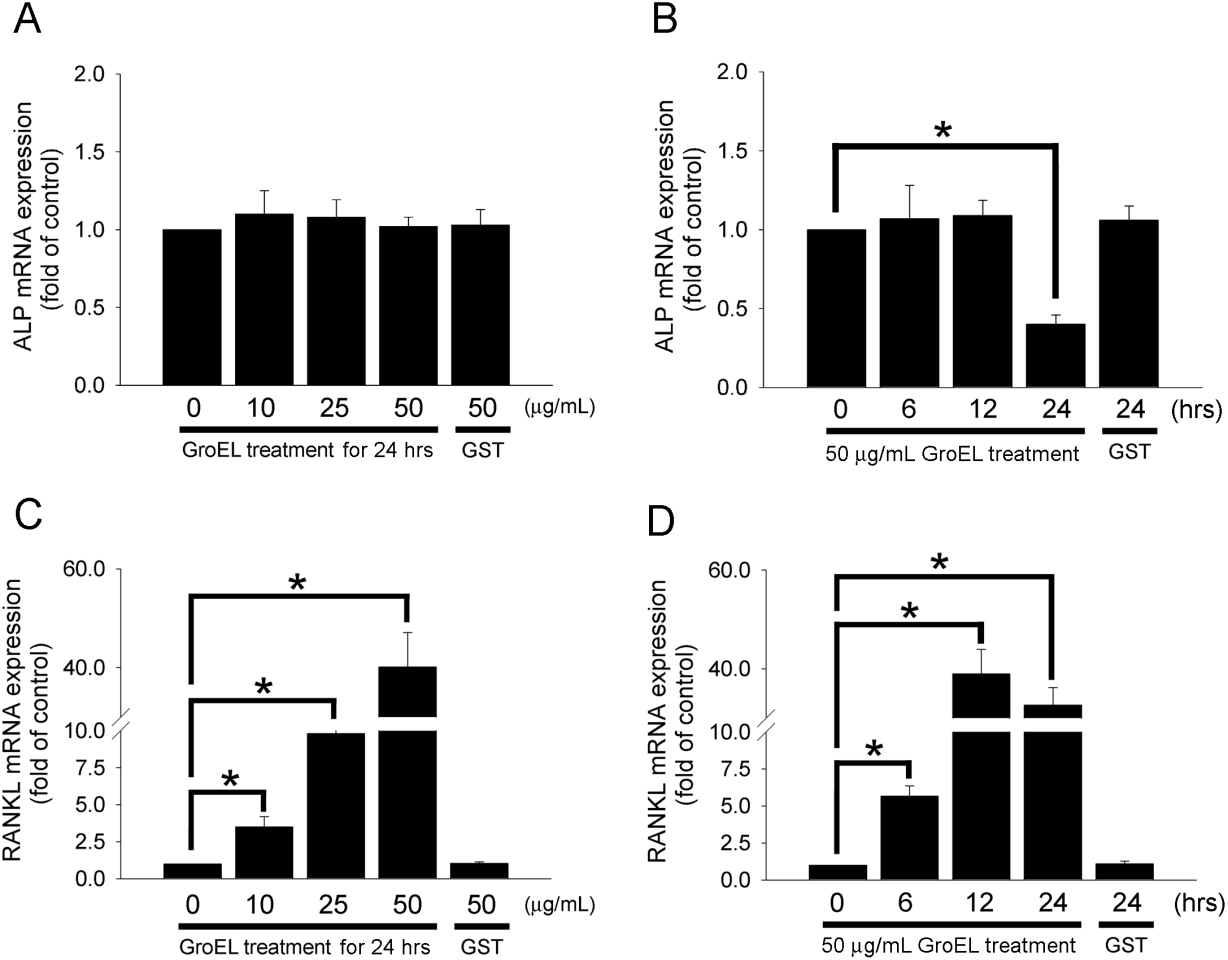
*P. gingivalis* GroEL may be involved in osteoclastogenesis via RANKL activation and ALP inhibition in PDL cells. (A and C) PDL cells were treated with serum-free media containing 0–50 µg/mL GroEL or 50 µg/mL GST for 12 h. (B and D) PDL cells were treated with 50 µg/mL of GroEL for 0–24 h or 50 µg/mL of GST for 24 h. The levels of ALP and RANKL mRNA were quantified using quantitative real-time PCR. (E) PDL cells were treated with 50 µg/mL GroEL or 100 µM pyrrolidine dithiocarbamate (PDTC) plus 50 µg/mL GroEL for 6 h. The levels of RANKL mRNA were quantified using quantitative real-time PCR. Data are expressed as the mean ± SEM of three experiments performed in triplicate. *p<0.05 compared with control cells (0 µg/mL GroEL).

Although the results indicated that the treatment of PDL cells with 10–50 µg/mL of GroEL for 12 h did not change ALP mRNA expression levels (figure 3A), treatment with 50 µg/mL of GroEL for 24 h significantly decreased ALP mRNA production by 0.4±0.06-fold (figure 3B). In addition, GroEL treatment significantly induced RANKL mRNA expression in a dose- and time-dependent manner. The treatment of PDL cells with 10, 25 or 50 µg/mL of GroEL for 12 h increased RANKL mRNA expression by 3.5±0.7-fold, 9.8±0.9-fold, or 40.1±7.0-fold, respectively (figure 3C). Treatment with 50 µg/mL of GroEL for 6, 12 or 24 h increased RANKL mRNA expression by 5.7±0.7-fold, 39.0±5.0-fold, or 32.7±3.6-fold, respectively (figure 3D). Additionally, pretreatment with 100 µM of PDTC for 30 min may decrease the RANKL mRNA production in GroEL-stimulated PDL cells (figure 3E). These results suggest that *P. gingivalis* GroEL can increase RANKL mRNA production via NF-κB activation, promote osteoclastogenesis via RANKL activation, and inhibit osteoblastic activity through the downregulation of ALP in PDL cells.

### *P. gingivalis* GroEL-induced Bone Loss in Rat Gingiva

**Figure 4:**
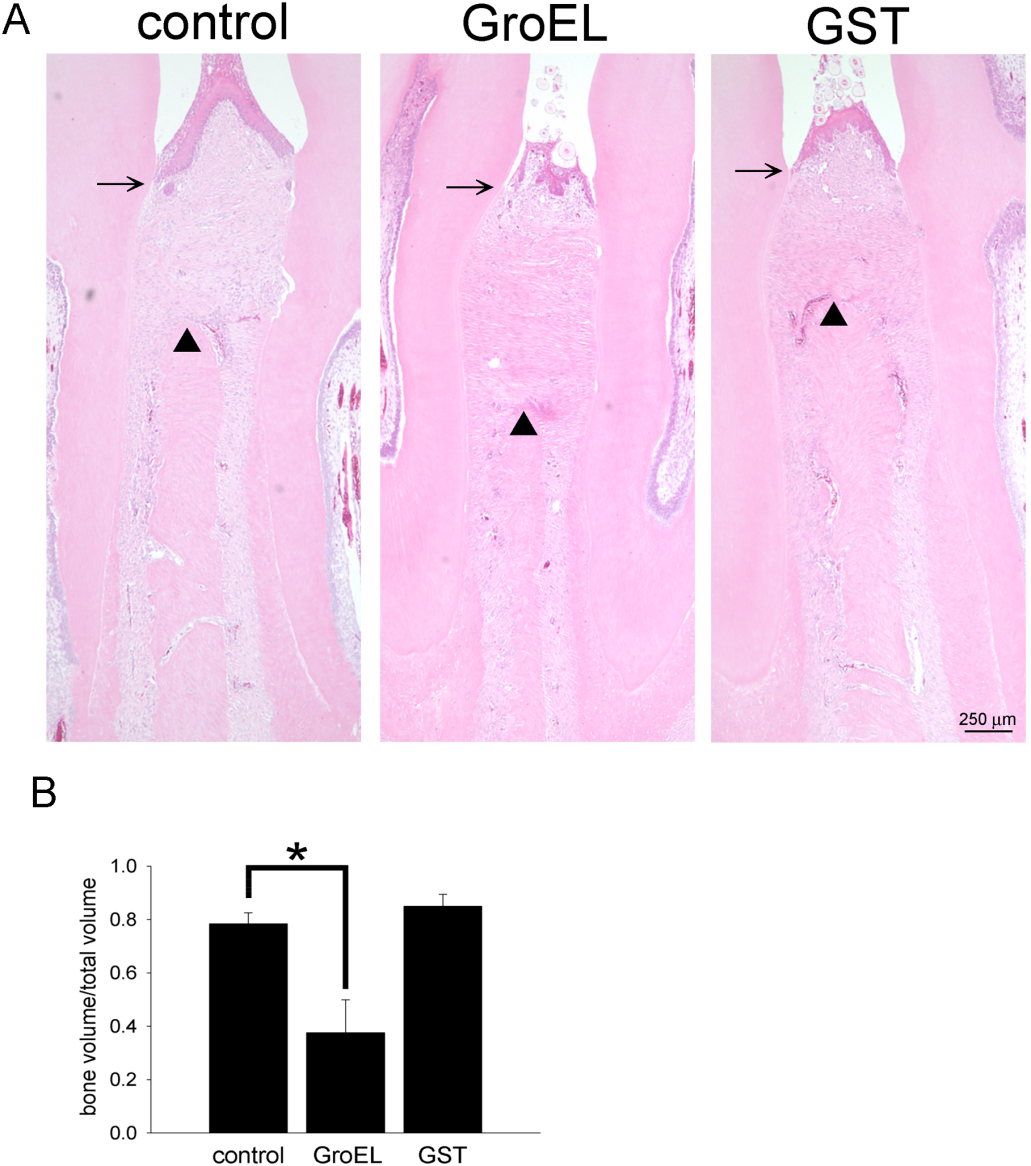
*P. gingivalis* GroEL induces bone loss in rat gingival. (A) Maxilla sections were stained using H&E. The black arrow indicates the cemento-enamel junction (CEJ) and the triangle arrowhead indicates the alveolar bone. (B) The specimens were scanned by micro-CT at a resolution of 35 µm, and the results were quantified as the ratio of residual bone volume (BV) per total volume (TV). The data represent the mean ± SEM of four animals (n = 4). *p<0.05 indicated a significant difference compared with the PBS injection control.

**Figure 5:**
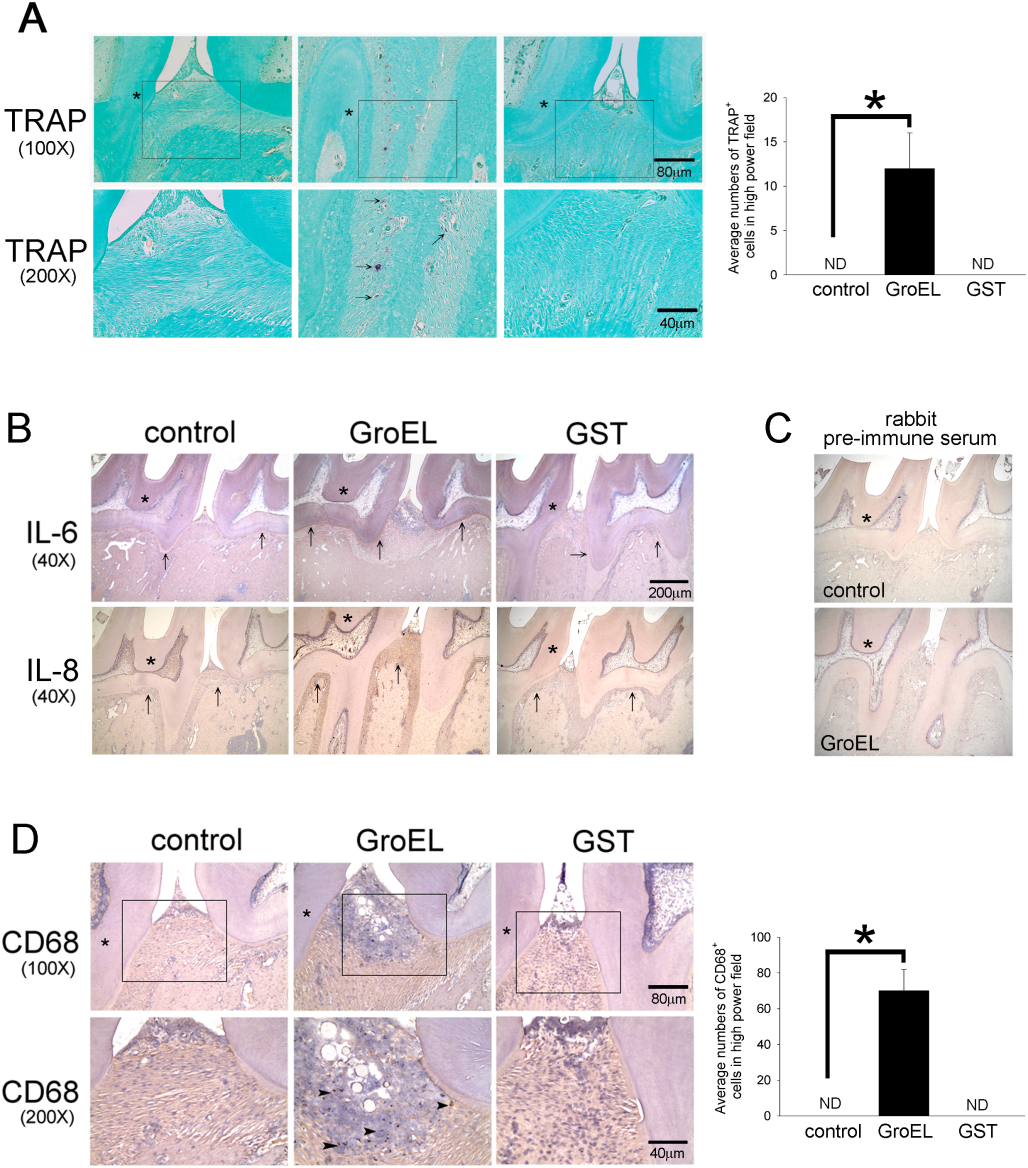
GroEL induces cytokines expression and macrophages as well as osteoclasts infiltration in rat gingival. (A) TRAP histochemistry was used to identify active osteoclasts in maxilla sections. The first molar is labeled by the star symbol, and the black arrows indicate the osteoclasts. (Lower panel) The image highlights a high-power view (200x) of osteoclasts in the connective tissues isolated from the images in the middle panel. The right graphs show the quantitative data of TRAP-positive cells in high-power field (HPF, magnification of 200x). Data are expressed as the mean ± SEM of three slides. *p<0.05 compared with control cells. (B) The expression of IL-6 and IL-8 were stained using specific antibodies and hematoxylin. The periodontal membrane (periodontal ligament) is indicated with the black arrow. (C) Negative control analyses were performed using rabbit pre-immune serum to replace specific antibodies. (D) Representative images (40x) of infiltrated macrophages stained with anti-CD 68 in the gingiva. The black arrowheads indicate the infiltrated macrophages. The right graphs show the quantitative data of CD68-positive cells in HPF (magnification of 200x). Data are expressed as the mean ± SEM of three slides. *p<0.05 compared with control cells.

*P. gingivalis* GroEL was injected between the left maxillary first and second molars of rats, and the effect on periodontal disease was subsequently examined. H&E staining and histological analysis demonstrated that the distance between the cemento-enamel junction and alveolar crest height (CEJ-AC) was greater in the GroEL-injected group relative to GST and control (PBS) groups at 6 weeks, indicating that treatment with GroEL caused periodontal tissue destruction (figure 4A). When the specimens were examined by micro-CT analysis, the data showed that injection of GroEL caused a decrease in the bone volume (BV)/total volume (TV) ratio (0.39±0.14) compared with the PBS (0.79±0.04) or GST (0.87±0.04) control groups (figure 4B), indicating that injection of GroEL led to higher bone loss relative to the control groups. Injection of GST did not significantly change the ratio, indicating that the effect was indeed caused by *P. gingivalis* GroEL. Additionally, the immunohistochemistical photos showed GroEL significantly induced occurrence of TRAP-positive osteoclasts to the gingiva (figure 5A, marked by black arrows). These results demonstrate that *P. gingivalis* GroEL itself has the ability to cause periodontal tissue disruption and is a potent virulence factor in *P. gingivalis*-mediated periodontal disease progression.

### GroEL-induced IL-6 and IL-8 Expression as well as Macrophage Infiltration in Rat Gingiva

To assess the impact of *P. gingivalis* GroEL on the inflammatory response in gingival tissue, we examined both the expression of IL-6 and IL-8 and the relative number of infiltrated macrophages in histopathological sections using immunocytochemical techniques. As shown in figure 5B, there was little IL-6 and IL-8 expression in the area of the periodontal membrane (periodontal ligament) in the control and GST treatment groups, as indicated by the black arrows. In contrast, the injection of GroEL induced significant expression of IL-6 and IL-8 in the periodontal membrane area of the rat gingiva. As compared to the control and GST treatment groups, GroEL significantly induced infiltration of CD68-positive macrophages to the gingiva (figure 5D, marked by black arrows). These results indicate that GroEL injection can potentially induce a chronic inflammatory response in the rat gingiva.

## Discussion

To the best of our knowledge, this is the first study demonstrating the effect of *P. gingivalis* GroEL on human PDL cells *in vitro* and on alveolar bone desorption in rats *in vivo.* In this study, treatment with *P. gingivalis* GroEL increased the production of the bone resorption-associated cytokines IL6- and IL-8, potentially via NF-κB activation, in PDL cells. *P. gingivalis* GroEL may also induce the overexpression of integrin α1 and α2, as well as cytoskeletal reorganization, to effectively stimulate PDL cell migration. Further, *P. gingivalis* GroEL stimulated the osteoclastogenesis-promoting functions of PDL cells, increasing RANKL expression and inhibiting ALP expression. Our *in vivo* results demonstrated that the injection of *P. gingivalis* GroEL caused alveolar bone loss in rats by micro-CT analysis. Immunohistopathological staining of sections further showed increased IL-6 expression and macrophage infiltration in GroEL-injected rat gingiva. These results strongly suggest that *P. gingivalis* GroEL itself has the ability to activate an inflammatory response, and can act as a potent virulence factor in *P. gingivalis*-induced periodontal disease progression.

*P. gingivalis* is frequently found in the subgingival flora of periodontitis patients and contributes to periodontal disease pathogenesis [32]. Cell surface components of *P. gingivalis*, such as lipopolysaccharide (LPS) and fimbriae, are potent stimulators that induce production of inflammatory cytokines and bone resorption [33]–[35]. *P. gingivalis* GroEL belongs to a highly conserved family of cytoprotective cellular proteins that are produced in response to a variety of environmental challenges. A previous study [36] has reported that *P. gingivalis* produces the HSP60 stress protein when subjected to different environmental stresses, suggesting that *P. gingivalis* HSP60 may also act as a stimulator of periodontitis. Researchers have also recently speculated that the immune response to different stress proteins initiates an inflammatory response, which may eventually lead to a chronic inflammatory state. In this study, both our *in vitro* and *in vivo* results strongly indicate that *P. gingivalis* GroEL functions as a virulence factor and has the ability to activate an inflammatory response in periodontitis. Bacterial HSPs have been reported to activate the production of pro-inflammatory cytokines [12]–[14], [37] and the upregulation of adhesion molecules [15], [16] in human monocytes. Consistent with this finding, our ELISA data showed that *P. gingivalis* GroEL treatment of PDL cells caused increased IL-6 and IL-8 production, possibly via NF-κB activation, and stimulated cell migration via integrin α1 and α2 activation and cytoskeletal reorganization. In 2002, Ueki *et al.* [11] demonstrated that recombinant human HSP60, but not *P. gingivalis* and *A. actinomycetemcomitans* GroEL (10 µg/mL), stimulated tumor necrosis factor-α (TNF-α) production in phorbol myristate acetate (PMA)-stimulated THP-1 cells. This observation suggests that the immune response to endogenous HSP60 produced by inflammatory tissues plays a role in periodontitis. In contrast, in our study, 10 µg/mL, 25 µg/mL and 50 µg/mL treatment with recombinant *P. gingivalis* GroEL increased the production of IL-6 and IL-8 in PDL cells. Argueta *et al.* have demonstrated that recombinant *P. gingivalis* GroEL (10 µg/mL) is also able to stimulate the transcriptional activity of NF-κB via the TLR2 or TLR4 receptors using luciferase reporter assays in THP-1 cells [20]. Although the specific receptor(s) that mediate the initiation of GroEL signaling in PDL cells remained to be determined, based on previously published work, we suggest that *P. gingivalis* GroEL may activate the same intracellular signaling cascade, resulting in NF-κB activation and gene activation in PDL cells.

Alveolar bone formation and resorption are regulated in part by cytokines released by PDL cells [38]. Furthermore, PDL-derived cytokines are sufficient to stimulate the migration of osteoclast precursors from the bone marrow to the periodontal space, where they differentiate into mature osteoclasts and absorb alveolar bone [39]. The membrane-bound protein RANKL was found to be expressed on the surface of osteoblasts, stromal cells, and PDL cells [26]. In addition, osteoclast precursors and osteoclasts have been found to express the RANKL receptor, which is responsible for transducing the RANKL signal [40]. Osteoprotegerin (OPG), a soluble tumor necrosis factor receptor homolog, has been found to inhibit osteoclast differentiation by competing with the binding of RANKL to the RANKL receptor [41], [42]. Thus, the cytokines expressed by PDL cells play important roles during osteoclastogenesis. It has been reported that PDL cells regulate osteoclast differentiation through dual regulatory mechanisms, stimulating RANKL expression and inhibiting OPG [26]. In our study, the treatment of PDL cells with *P. gingivalis* GroEL induced RANKL expression and inhibited the expression of ALP, a conserved factor involved in the calcification of various mineralizing tissues that has been used as an indicator of osteoblastic activity in bone tissue. These data suggested that GroEL may affect not only the activation of osteoclast differentiation but also the inhibition of osteoblastic activity. Previous studies have shown that hormones such as parathyroid hormone (PTH), 1,25-dihydroxy vitamin D3, and prostaglandin E_2_ (PGE_2_) [43], as well as cytokines such as TNF-α, interleukin (IL)-1β, and IL-6 all have the potential to induce the expression of RANKL [44]. *P. gingivalis* is known to induce RANKL expression in osteoblasts and PDL cells [45], [46]. In our results that treatment with *P. gingivalis* GroEL induced not only IL-6 and IL-8 but also RANKL production. RANKL mRNA expression significantly increased within 6 hrs after 50 µg/mL *P. gingivalis* GroEL treatment. Pretreatment with 100 µM of PDTC for 30 min may decrease the RANKL mRNA production in GroEL-stimulated PDL cells, suggested that *P. gingivalis* GroEL can increase RANKL mRNA production via NF-κB activation. The other scientists supported that RANKL expression is regulated by c-Fos through a cluster of distal regulatory enhancers in T cells [47]; histone deacetylase inhibitors may increase endogenous expression of RANKL resulting from enhanced acetylation of histones on the proximal RANKL promoter [48]. Although it has been shown that IL-6 can stimulate bone resorption by its ability to up-regulate RANKL in osteoblast [49], IL-6 seems to do not have effects on RANKL production in PDL cells [50]. In previous report, *P. gingivalis* culture supernatant was sufficiency to induce RANKL mRNA expression within 6 hrs after challenge [45] further support that some virulence factors have direct effect on RANKL production in PDL cells. In addition to NF-κB, whether other factors direct to involve in the expression of RANKL in *P. gingivalis* GroEL-stimulated PDL cells is remain to be elucidated.

Additionally, bacterial LPS components have strong bone resorption activity. *A. actinomycetemcomitans* GroEL acts as a potent bone resorption factor in a murine calvarial resorption assay [51]. Here, we also confirm that *P. gingivalis* GroEL can induce bone resorption in rats and may also act as a bone resorption factor, in addition to LPS. The specific intracellular signaling pathways that mediate the *P. gingivalis* GroEL-induced effects will need to be clarified in the further studies. We strongly suggest that in periodontal disease progression, *P. gingivalis* GroEL itself can act as a virulence factor in addition to LPS and fimbriae, two well-known virulence factors in *P. gingivalis*.

Previous evidences had demonstrated that the network of cytokines and chemokines, production by resident cells (epithelial cells, gingival fibroblasts, PDL cells, osteoblasts, and dendritic cells) as well as migrating cells (lymphocytes and phagocytes), involved in periodontal bone resorption. For example, epithelial cells and gingival fibroblast are responsible for the production of IL-8, which recruits neutrophils and increases monocytes adhesion; in turn can differentiate into osteoclasts [52]. In addition, both gingival fibroblasts and periodontal ligament fibroblasts make response to product IL-6 which further stimulates bone resorption by its ability to up-regulate RANKL, a proosteoclastogenic cytokine, expression in osteoblasts and PDL cells [53]. IL-8 and IL-6 are directly or indirectly involved in osteoclastogenesis, and are responsible for the alveolar bone loss in periodontitis. It was reported that IL-6 levels in inflammatory gingival tissue were higher than those in health control tissue [54]. However, a range of proinflammatory cytokines and chemokines such as IL-1α, IL-1β, TNF-α are also reported to be responsible for these process, which may initiate connective tissue inflammation and alveolar bone resorption [55]. Although our results showed bone loss and increased IL-6 and IL-8 expression in GroEL-injected rat gingival, it still can not provide direct evidence that IL-6 and IL-8 are really involved in the GroEL-induced alveolar bone loss, and can not measure whether GroEL induces other major bone resorption-activating factors. Indeed, the direct roles of IL-6 and IL-8 in GroEL-induced alveolar bone loss in animal and the effects of P. gingivalis GroEL on other major bone resorption-activating factors should be determined and clarified in further studies.

In Figure 2A and B, we demonstrated that *P. gingivalis* GroEL increased PDL cell migration. Even though periodontal fibroblast migration plays an essential role in periodontal wound-healing process, we still speculate that enhanced migration ability in PDL cells promoted the coordination between other periodontal resident cells including epithelial cells, gingival fibroblast, osteoblast, and dendritic cells which corporately mediate the innate immunity in the initiation stage of periodontitis. Indeed, in figure 5A showed that GroEL significantly induced occurrence of TRAP-positive osteoclasts to the gingival. It suggests that *P. gingivalis* GroEL has the ability to cause PDL cells infiltration and increase osteoclastogenesis in rat maxilla.

Cell migration is a complex phenomenon which is mediating by multiple regulatory process. Integrins are transmembrane proteins that mediate the attachment between a cell and its surroundings (including other cells or the extracellular matrix).

The intracellular domain of integrin is also associated with cytoskeleton/actin, and regulates the cell migration [56]. Additionally, integrins may involve in the regulation of cell signaling, cell cycle, and cell motility [57]. Collagen is one of the main components in periodontal extracellular matrix which maintains the organization of structure and provides an scaffold for tissue repair [58]. Integrins α1β1 and α2β1 are the receptors for collagen [59]. Integrin α1β1 mainly interacts with type IV collagen and Integrin α2β1 interacts with type I collagen [60], [61] which are involving in the migration of PDL cells [62]. In this study, GroEL from *P. gingivalis* GroEL may induce the PDL cells migration, and the real-time PCR indicated that GroEL may increase the expression of integrins α1 and α2 in PDL cells. Therefore, we predicted that GroEL may induce the PDL cells migration mediating by the expression of integrins α1 and α2. Additionally, previous evidences showed integrins α10β1 and integrins α11β1 regulate human PDL cells activation [62], [63]. Whether *P. gingivalis* GroEL affects the expression of integrins α10β1 and α11β1 remain to be elucidated.

In conclusion, this report represents the first direct evidence suggesting a possible role for *P. gingivalis* GroEL in inflammatory periodontal disease, demonstrating that proinflammatory cytokines are directly induced and factors regulating bone remodeling are regulated in GroEL-treated PDL cells. This information will, in turn, lead to the development of new therapeutic strategies for controlling periodontal disease.

## Limitation

In figure 5C (CD68 staining), round shaped structures in the area of hematoxylin-stained blue area were noted. We speculated that the round shaped structures may be resulting from tissue apoptosis or necrosis. In general, the voids in apoptotic or necrotic tissue may be filled by droplets, and show as a circular structures after immunohistochemistry. In this study, we mainly investigated the effect of GroEL on human PDL cells, as well as its effect on alveolar bone resorption. Hence, we can not determine whether administration of GroEL may induce gingival necrosis or apoptosis in the absence of analysis. We do not exclude the possibility that GroEL may cause apoptosis or necrosis in gingival, and we will elucidate the issue in the further.
